# Evaluation of word embedding models to extract and predict surgical data in breast cancer

**DOI:** 10.1186/s12859-022-05038-6

**Published:** 2022-11-16

**Authors:** Giuseppe Sgroi, Giulia Russo, Anna Maglia, Giuseppe Catanuto, Peter Barry, Andreas Karakatsanis, Nicola Rocco, ETHOS Working Group, Francesco Pappalardo

**Affiliations:** 1 Department of Mathematics and Computer Science, University of Catania, 95125 Catania, Italy; 2 Department of Drug and Health Sciences, University of Catania, 95125 Catania, Italy; 3 G.RE.T.A. Group for Reconstructive and Therapeutic Advancements, Catania, Italy; 4 Multidisciplinary Breast Unit, Azienda Ospedaliera Cannizzaro, Catania, Italy

**Keywords:** Machine learning, Word embeddings, Word2Vec, Breast cancer, Natural language processing

## Abstract

**Background:**

Decisions in healthcare usually rely on the goodness and completeness of data that could be coupled with heuristics to improve the decision process itself. However, this is often an incomplete process. Structured interviews denominated Delphi surveys investigate experts' opinions and solve by consensus complex matters like those underlying surgical decision-making. Natural Language Processing (NLP) is a field of study that combines computer science, artificial intelligence, and linguistics. NLP can then be used as a valuable help in building a correct context in surgical data, contributing to the amelioration of surgical decision-making.

**Results:**

We applied NLP coupled with machine learning approaches to predict the context (words) owning high accuracy from the words nearest to Delphi surveys, used as input.

**Conclusions:**

The proposed methodology has increased the usefulness of Delphi surveys favoring the extraction of keywords that can represent a specific clinical context. It permits the characterization of the clinical context suggesting words for the evaluation process of the data.

**Supplementary Information:**

The online version contains supplementary material available at 10.1186/s12859-022-05038-6.

## Background

In about 2/3 of the cases, healthcare decisions are insufficiently relying on data PricewaterhouseCoopers (PWC) [1]. Some studies highlight that cognitive shortcuts, called heuristics, are relevant in medical practice [2, 3].

Trials, when available, include only a narrow population (< 1% for solid tumors [4]) with significant disparities in race, age, stage, and organ involvement. In breast cancer surgery (BCS), a few systematic reviews performed by our team [5–9], have confirmed a generalized poor quality of information available.

For instance, the recent GRADE-based metanalysis comparing standard breast-conserving surgery to the oncoplastic-based techniques has revealed a low level of evidence with a lack of a randomized trial and absence of standard tools for evaluation of clinical outcomes. Surprisingly, despite the substantial controversy, about one-third (36%) of panel members expressed a strong recommendation supporting oncoplastic BCS [5].

Similarly, a Cochrane systematic review revealed that despite a central role of implants for breast reconstruction, these had been studied rarely in the context of Randomized Controlled Trials (RCTs). Thus, a few million women undergo breast reconstruction without adequate information about risks and complications [7].

Therapeutic resolutions about breast cancer surgical management have become rather intricate over the last 20 years. Patients affected by early-stage breast cancer may have up to 2592 possible combinations in front of them depending on disease characteristics, breast volume and shape, and patients' preferences. A specific tool has been designed to navigate the final oncoplastic decision [10]. Despite being based on fragmented information, shared decision-making played a determinant role in simplifying the process and de-escalation of complexity [11].

In this scenario, decisions at the patient level are approximated by doctors' intuition and rely primarily on personal judgment.

Structured interviews denominated Delphi surveys have been designed to investigate experts' opinions and solve by consensus complex matters like those underlying surgical decision-making. There are no defined methodologies for Delphi questionnaires, but in the past, in the first round, it has been advised to let the information flow without constraints using narrative interviews [12]. This was our preferred strategy considering the demonstrated inconsistency of available reports and contrary to procedures recently proposed [13] based on formal analysis of existing evidence.

The primary endpoint of this study is to assist in the extraction of relevant features related to patients and disease characteristics, surgical techniques, and relevant outcomes from a list of unstructured interviews (ETHOS Delphi Survey). For this purpose, we gathered a global panel of world-leading experts that participated in the online process.

The second endpoint of this work is creating a tool to generate verbal harmonization of narrative databases of health electronic records (HER) in this context (e.g., clinical notes, theatre reports, nurse's notes, outpatient notes, etc.). Narrative data provide a considerable amount of verbally heterogeneous information that otherwise could be lost [14].

Natural Language Processing (NLP) is a field of study that combines computer science, artificial intelligence, and linguistics [15, 16]. As we know, managing or understanding human language is particularly complex for computer algorithms. The simple knowledge of the meaning of each word is not sufficient to correctly interpret the message of the sentence. On the contrary, it can lead to contradictory and meaningless communications. Research in this area has focused on the mechanisms that allow people to understand the content of human communication and the development of tools that can provide computer systems with the ability to understand and process natural language. NLP-processing involves a succession of steps that attempt to overcome the ambiguities of human language. In particular, it is a delicate process due to the complex characteristics of language itself. The processing is subdivided into several steps to reduce the number of errors as much as possible (e.g., tokenization, stemming, and lemmatization).

Nowadays, we often find the association between NLP and Machine Learning (ML). ML and NLP are concepts of an entirely different level, the former referring to a type of approach, while the latter represents a subject area. In reality, of course, machine learning goes far beyond the scope of NLP. Machine learning algorithms used for different cases of language processing can equally be used to solve other Artificial Intelligence (AI) problems, such as DNA sequence classification or medical diagnosis.

In 2020, an important milestone was reached in the world of natural language interpretation. OpenAI, a non-profit organization for artificial intelligence research, has released its latest language model based on neural networks called Generative Pre-trained Transformer 3 (GPT-3). To date, it is the most parameter-based network ever trained [17].

## Implementation

**Fig. 1:**
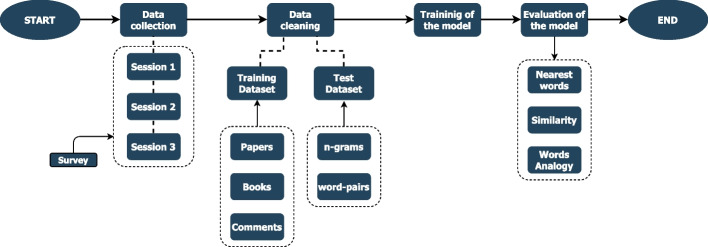
Functional scheme. The study consists of several steps: from data collection to various tests to evaluate the model

The workflow carried out in this study is illustrated in Fig. 1. Firstly, a data collection phase was conducted among the participating experts and consisted of three sessions. After receiving the data, a data cleaning phase was applied, leading to the creation of two datasets (training and testing). Finally, a Word2Vec neural network was trained, and the model was tested with standard evaluators.

In March 2021, a core team made up of senior oncoplastic breast surgeons (named "facilitators") affiliated to Group for Reconstructive and Therapeutic Advancements (G.RE.T.A.) met to plan study design and identified criteria for participation as follows:

- Senior members of National and International Societies
- Senior author of peer-reviewed papers on surgical decision making of breast cancer

An invitation to apply was published for one week on the website and social media of the organization (https://greta.maurizionava.it/). About 30 individuals replied, 26 were approved. The facilitator's team approved a list of questions. These were related to patients and disease characteristics and clinical outcomes. Participants were also invited to assess a list of surgical techniques and add any missing strategy. We performed three subsequent sessions of interviews between March 2021 and June 2021. A collection of books edited or co-edited by the participants was used to train the artificial neural network and a list of peer-reviewed papers was gathered by one of the facilitators.

### Data collection

**Fig. 2:**
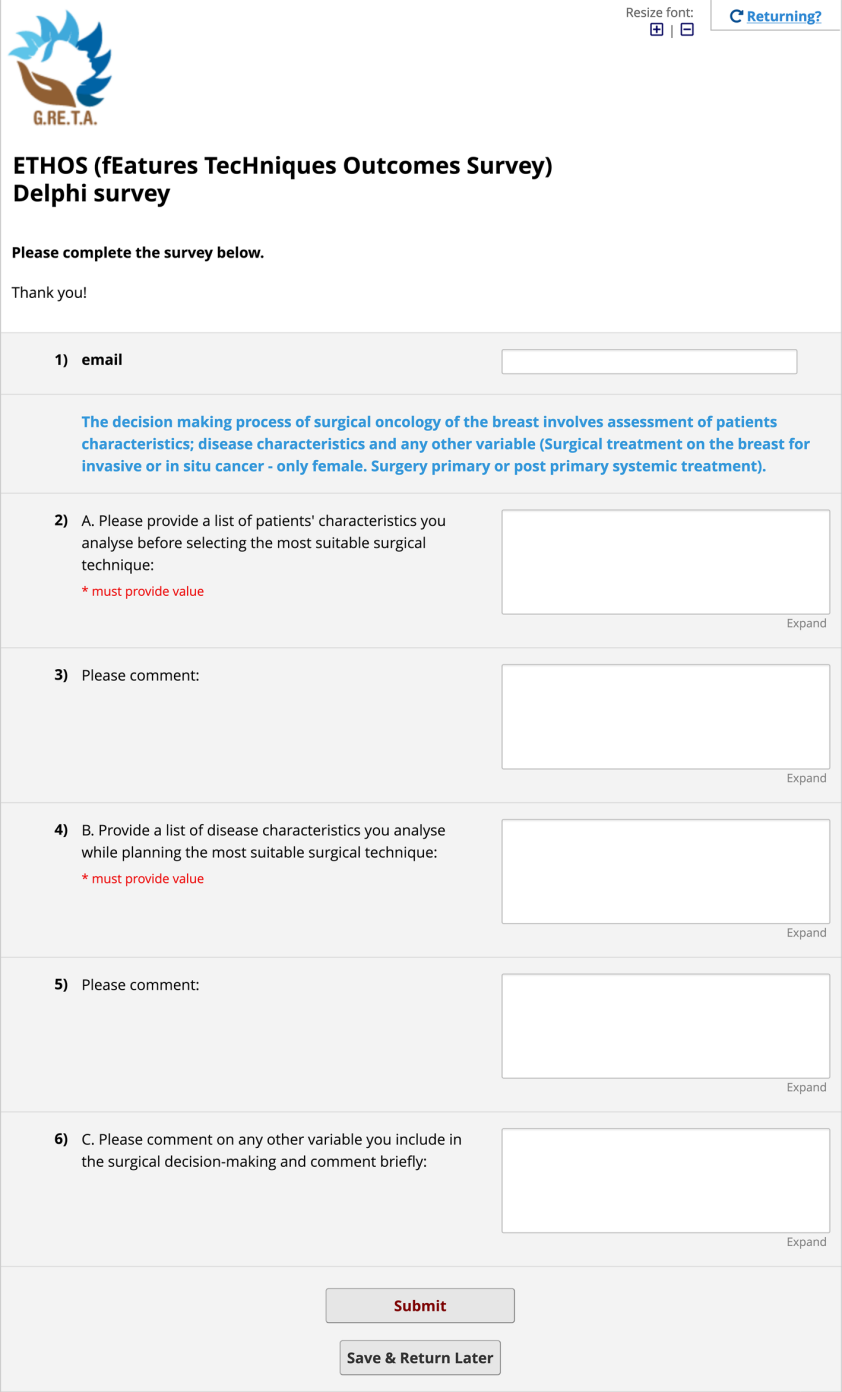
Session 1 survey. The figure represents the boxes of session 1 in which one is asked to list the characteristics of the patient and the disease and comment on them

**Fig. 3:**
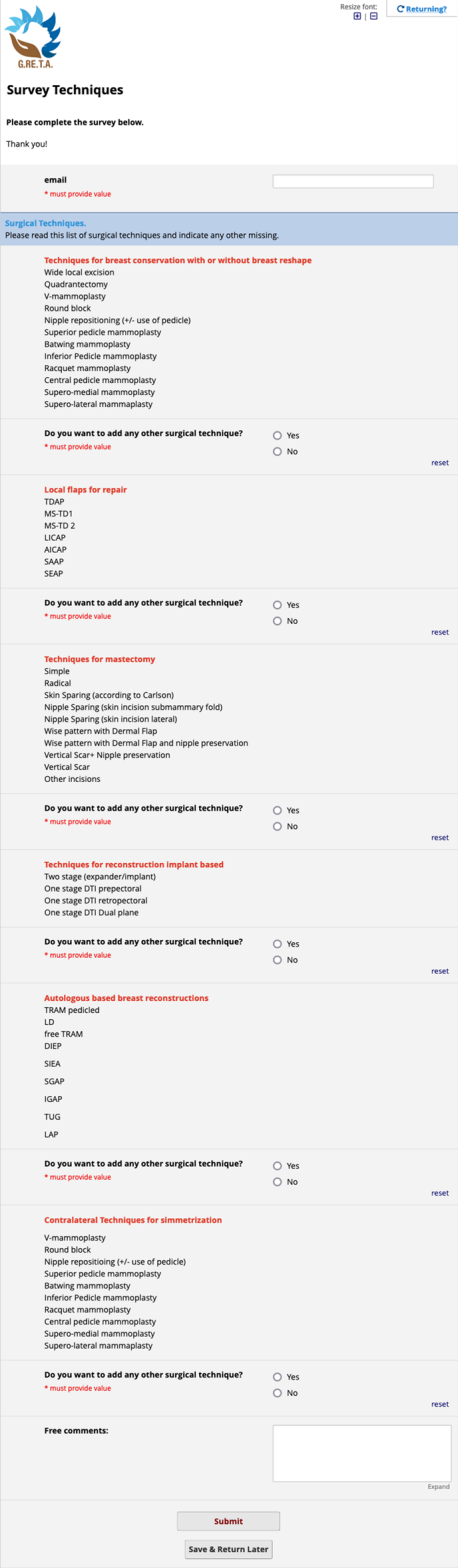
Session 2 survey. The figure depicts the questions in session 2 for each category of surgical techniques and comments on them

**Fig. 4:**
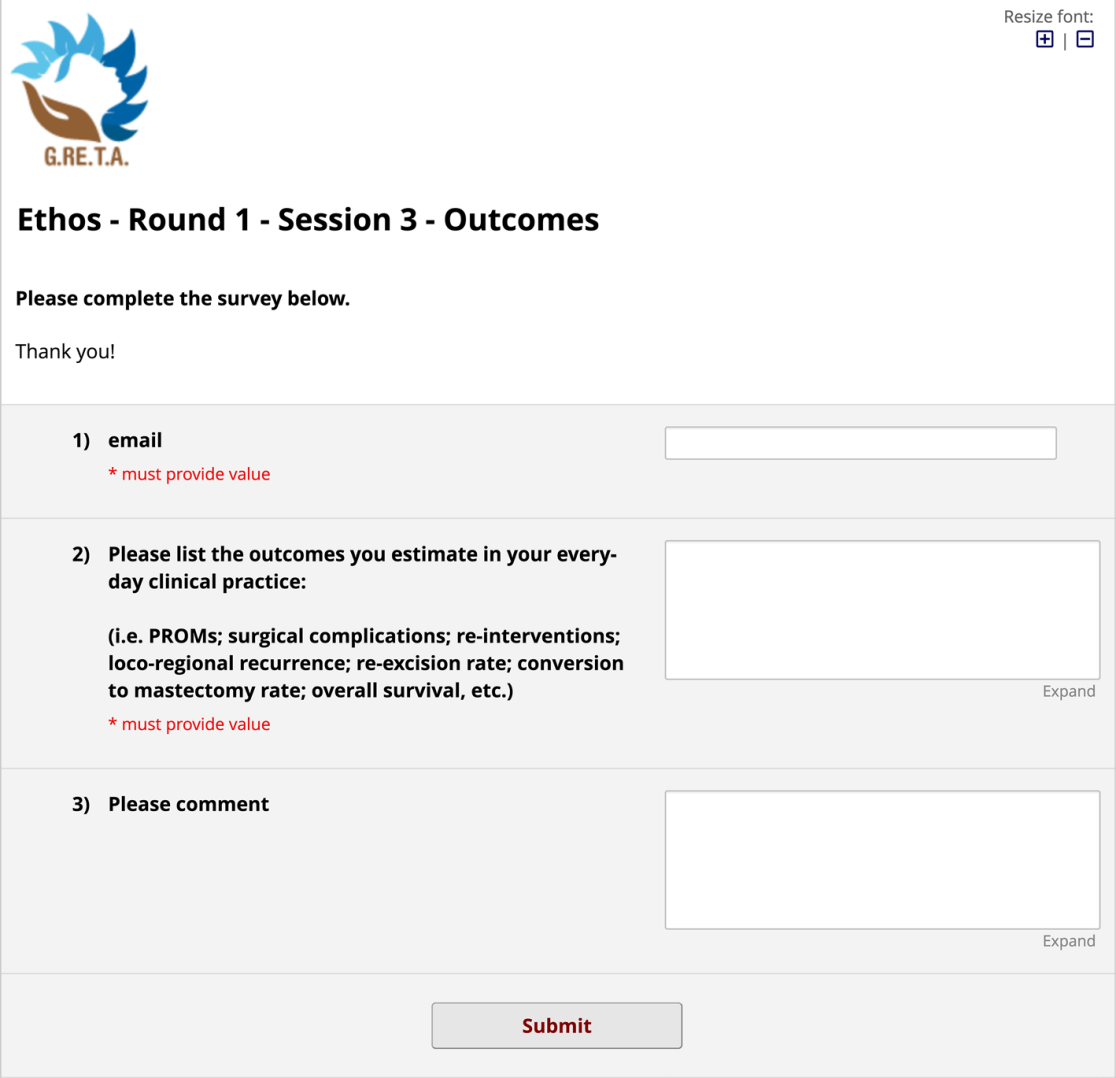
Session 3 survey. The figure shows the demands of session 3. It represents the opportunity to list the estimated results in daily clinical practice and to comment on them

Survey sessions were built and administered through a web application called REDCap,^1^ born for Survey design and Electronic Data Capture. Through this web designer tool, the three sections of the first round of interviews were created as follows:

- *Session 1* a questionnaire form made of six fillable note boxes, named ETHOS (fEatures TecHniques Outcomes Survey) Delphi survey, to enlist patient and disease characteristics along with related comments, and any other variable needed to be taken into account (Fig. 2).
- *Session 2* a questionnaire form made of fourteen fillable note boxes, named Survey Techniques, to retrieve for each category of surgical techniques (conservative, flaps, mastectomy, implant or autologous-based reconstruction and symmetry) a list of techniques to be approved. For each of them, either approval or specification was asked through a branching logic—conditional—structure of the fields (Fig. 3).
- *Session 3* a questionnaire form made of three fillable note boxes, named Ethos—Round 1—Session 3—Outcomes, to enlist everyday clinical practice outcomes and related comments; (Fig. 4)

REDCap meets the need to let only the selected experts participate in the questionnaire, allowing a participant list. Automatic invitations were sent at the pre-established time: March 15th for round 1—session 1, May 15th for round 1—session two and June 12th, 2021 for round 1—session 3. A reminder was sent once a week for those not answering at the first call. The response rate was 96% for sessions 1 and 2 and 77% for session 3. Round1 is composed of 3 sessions: one for patient and disease characteristics, one for surgical techniques and a third for outcomes. As mentioned, a second round is planned to be held soon. At the end of it, it is foreseen that only variables reaching a 75% consensus threshold will be accepted.

### Data cleaning

Data cleaning (DC), also known as data scrubbing, broadly refers to the process developed to help in removing errors from the reference text. Typically, DC consists of different steps: identifying, deleting, replacing incomplete, inaccurate, irrelevant portions of the text, or other issues related to.

The data collected in the previous step were processed through the following steps for each type of text (papers, books, or survey comments):

- Removal of punctuation and white spaces;
- Tokenization phase that consists in splitting the text into specific words made of at least three characters;
- Lemmatization: the process of converting a word into its base form. In other words, this method would correctly identify the base form of "caring" to "care". Also, sometimes, the same word can have multiple different lemmas. So, based on the context of use, lemmatization identifies the part-of-speech (POS) tag for that word in its specific context and extracts the appropriate lemma.
- Translation of the words from American to the English language;
- Data storage: if the final result is an empty list, this latter will not be included in the training or test dataset.

Steps 1–3 are applied through the spaCy library [18]. spaCy is a free, open-source library for NLP in Python. It is written in Cython and designed to build information extraction or natural language understanding systems. spaCy provides a concise and user-friendly API.

In conclusion, we generated two datasets, one to train the neural network and another one to evaluate the model. The training set consists of papers, books, and comments from the survey sessions. The test dataset was generated from the variables described by the various survey experts, and divided into i) n-grams and ii) word pairs. The n-grams are used to predict a context (or set of words) through closest words, while the word pairs are used to study their similarity.

### Training of the model

Word2vec (W2V) is a natural language processing technique. The word2vec algorithm uses a neural network model to learn word associations from a large corpus of text. Once trained, such a model can detect synonymous words or suggest additional words for a partial sentence. As the name suggests, word2vec represents each specific word with a particular list of numbers called vectors. The vectors are carefully chosen through a mathematical function (the cosine similarity between the vectors) that indicates the level of semantic similarity between the words represented by the vectors.

Training word embeddings involves the fitting of the model to a pre-processed corpus and the tuning of the model of the hyper-parameters, whose values are specified empirically before the training. Often, the performance increases with the size of the dataset up to a certain point.

W2V is a prediction-based method of word embedding that implements two different embedding methods: the Continuous Bag of Words (CBOW) model and the Skip-gram (SG) model [19–21].

Both the CBOW and SG models are examples of neural embedding models for learning the mapping of words to a specific point in the vector space [22]. These models employ shallow neural network architectures to understand the parameters of the embedding vectors. The difference between these methods is whether the neural network attempts to predict a focus word according to its context (CBOW) or the reverse (SG) one. Although the two models have similar architectures and approaches to parameter learning (i.e., stochastic gradient descent) [23], the specific loss functions are unique, reflecting the distinct objectives of the respective models.

In the CBOW model, the objective function involves the prediction of a focal word due to its context. The CBOW model is essentially a log-linear classification model with a multinomial/softmax loss function. The goal is to determine parameters of the embedding vectors that own a higher probability under the following formula:

$$P\left({\omega }_{f}|{\omega }_{c}\right)= \frac{\mathrm{exp}({\omega }_{f}^{T}{\omega }_{c})}{\sum_{i=1}^{V}\mathrm{exp}({\omega }_{i}^{T}{\omega }_{c})}$$

where ${\omega }_{f}$ is the focal word, ${\omega }_{c}$ is the context (one or more words), and $V$ is the vocabulary size. The hidden layer is merely the vector representation of the context word. The inner product between the context and the focal word vectors can be seen as an assigned score. Therefore, high scores map means high predicted probabilities by the model. The goal is to set high scores to focal words that are likely under the context.

The Skip-gram model is complementary to the CBOW model in the sense that its objective function involves the prediction of a context word(s), given a single focal word [24].

$$P\left({\omega }_{c}|{\omega }_{f}\right)= \sum_{c=1}^{C}\frac{\mathrm{exp}({\omega }_{c}^{T}{\omega }_{f})}{\sum_{i=1}^{V}\mathrm{exp}({\omega }_{c}^{T}{\omega }_{i})}$$

**Fig. 5:**
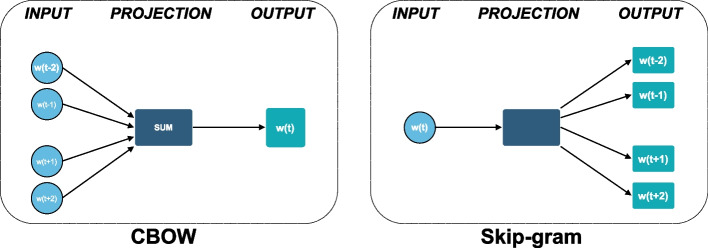
Model architectures. The CBOW architecture predicts the current word based on the context and the Skip-gram predicts surrounding words given the current word

In the case of a single-word context, the model is identical to CBOW. In the case of multi-word contexts, the objective becomes more different. Since there is only one word per context, the hidden layer copies the current vector representation of the focal word. However, the final objective aims at predicting C context words. Moreover, the model loss/objective function is a sum of the respective context-specific loss functions. In the end, C inner-product scores will be obtained, specifically one for each focal-word context-word pair. The goal will be to assign high scores to those context words likely under the given focal word (Fig. 5).

The word2vec algorithm includes skip-gram and CBOW models, using the hierarchical softmax (HS). One strategy is to reduce the computational complexity of a traditional softmax. The hierarchical softmax involves the replacement of the entire output layer with a binary tree whose leaves represent the words of the dictionary, and each node of the graph (not leaf) is associated with a word embedding that the model will learn.

The library that implements W2V and, in particular, CBOW and SG is called Gensim [25]. Gensim is an open-source Python library for natural language processing, with a specific focus on topic modeling. Gensim was developed by Czech researcher Radim Řehůřek (RaRe Technologies). In particular, Gensim is a mature, focused, and efficient suite of NLP tools for topic modeling. It supports the implementation of the Word2Vec word embedding for learning new word vectors from text and provides specific tools for loading pre-trained word embeddings in a few formats and for making use and querying a loaded embedding.

The four critical parameters for training Word2Vec embeddings are *i)* words below the minimum frequency are dropped before training occurs. Hence, the relevant context window is the word distance among surviving words. If the default minimum frequency is equal to 5, and a word only appears 4 or fewer times across all the documents, it will be ignored. Viceversa, if it appears 5 times in a single document, it will be considered; *ii)* the number of the embedding dimensions, typically between 50 and 500, are tuned experimentally); *iii)* the length of the context window (i.e., how many words before and after the target word are used within the context for training the word embeddings; it is worth mentioning that they are 5 or 10 words usually); *iv)* the number of epochs (i.e., hyperparameter that defines the number times that the learning algorithm will work through the entire training dataset) usually benefits the quality of the word representations. Therefore, more epochs could improve the results. There are other more advanced hyper-parameters for W2V. Training embeddings with more dimensions typically require more training data and more computational time. Each dimension should capture some aspect of meaning, so the embeddings need to be large enough to differentiate words.

In our case, the model was trained with different combinations of parameters. Table 1 shows the combinations of the tested hyper-parameters. At the same time, the results (word pairs) of these hyper-parameters combinations can be found in the Additional file 1: Supplementary data. Among all the obtained results, the only one (Test16) that returned the expected result is the one owing the following parameters: i) min_count = 5, ii) window = 20, iii) vector_size = 300, and iv) epochs = 100.

**Table 1:** Hyper-parameters of W2V

min_count	Window	vector_size	Epochs	Mode	Softmax
5	5	100 or 200 or 300	10	Skip-gram	enable
3	5	100 or 200 or 300	10 or 100	Skip-gram	enable
5	3	100 or 200 or 300	10	Skip-gram	enable
5	10	300	10	Skip-gram	enable
3	10	300	10	Skip-gram	enable
10	5	300	10	Skip-gram	enable
3	20	300	10	Skip-gram	enable
3	20	300	100	Skip-gram	enable
5	20	300	100	Skip-gram	enable
10	20	300	100	Skip-gram	enable

All combinations of parameters tested are shown. In this way, a correct model has been found that correctly represents our context

### Evaluation of the model

The evaluation of a W2V model depends on the purpose one considers for the word vectors. In particular, it should mimic the final use as much as possible [26]. The goal of an evaluator is to compare the characteristics of different word embedding models with a quantitative and representative metric. However, it is not easy to find a concrete and uniform way to evaluate these abstract characteristics. For example, other issues in the training phase could be present even if a hand-crafted evaluation set is of high quality for specific purposes, and the word-vectors are not performing well. This could depend on data availability, errors in the pre-processing phase, or a poor choice of the meta-parameters.

Usually, a good evaluator should focus on the following properties:

- *Good test data* To have a reliable representative score, test data should be varied with a good spread over space. All the words that recur frequently or rarely should be included in the assessment.
- *Completeness* An evaluator should test many properties of a word embedding model. This also allows determining the effectiveness of an evaluator.
- *High correlation* The score of a word model in an embedding assessment task should well correlate with the model performance in natural language processing tasks.
- *Efficiency* Evaluators should be computationally efficient. Model evaluators should be able to predict the downstream performance of a model in a simple way.
- *Statistical significance* The performance of different word embedding models concerning an evaluator should have statistical significance enough or enough variance between the scoring distributions to be differentiated [27].

These properties are needed to judge whether one model is better than another and help to determine the performance ranking among models. Furthermore, the absolute value of an evaluation score may not be relevant because it may not indicate the final goal in terms of words. Word-vectors might still work well enough in other fuzzier information-retrieval contexts.

A common mistake during both training and evaluation is to retain too many rare words. Words with only a few occurrences may not lead to very high-quality vectors. However, the final vectors of the most frequent words are strongly influenced by random initialization and not by their common meaning. Moreover, rare words presence may interfere with the improvement of the general context.

Word semantic similarity method is based on the idea that the distances between words in an embedding space could be evaluated through the human heuristic judgments on the actual semantic distances between these words. This method is one of the most popular evaluation methods nowadays.

The assessor is given a set of pairs of words. After, it is asked to assess the degree of similarity for each pair. The distances between these pairs are also collected in a word embeddings space, and the two obtained distances sets are compared. The more similar they are, the better are embeddings [28–33]. Over the years, several datasets have been created to check for word similarity. In particular, different datasets use different notions of lexical semantic similarity in such a way that the same embeddings may have different results. Below some datasets created to check for similarity are reported:

- *WordSim-353 (WS353)* 353 pairs assessed by semantic similarity with a scale from 0 to 10 [34, 35].
- *Rare Word (RW)* 2034 pairs of words with low occurrences (rare words) assessed by semantic similarity with a scale from 0 to 10 [36].
- *SimLex-999* 999 pairs assessed with a strong respect to semantic similarity with a scale from 0 to 10 [35, 37].
- *UMNSRS* consists of 449 clinical term pairs whose semantic similarity and relatedness were determined independently by four medical residents from the University of Minnesota Medical School [38, 39].

Another method, or rather the second most famous for evaluating word embeddings, is the Word analogy. It is based on the idea that arithmetic operations in a word vector space could be predicted by humans [28, 40, 41]. The aim of the word analogy is to try to complete such an expression:

$$A :B : :C : ?$$

To make an example, one has the following words A = Paris, B = France, C = Rome. Then the target word would be Italy since the relation A:B is capital:country, hence one needs to find the capital of which country is Rome.

Specifically, the model tries to predict a word D so that the associated word vectors A, B, C, D are related. After that, the measure of the similarity takes the vectors between B − A and D − C using cosine similarity. Given two numerical attribute vectors, A and B, the level of similarity between them is expressed using the formula:

$$similarity= \mathrm{cos}\left(\theta \right)= \frac{A\bullet B}{\Vert A\Vert \Vert B\Vert }$$

The similarity value thus defined is between − 1 and + 1, where − 1 indicates an exact but opposite match and + 1 indicates two equal vectors. Datasets designed for semantic relation extraction tasks could also compile a word analogy set [42]. Below a list of datasets that could be used for the evaluation of this method is proposed:

- *WordRep* about 118 billion analogy questions divided into 26 semantic classes. It is an extensive data set of Google Analogy with additional data from WordNet.
- *Google Analogy* 19544 questions are divided into two classes (morphological relations and semantic relations) and ten smaller subclasses (8869 semantic questions and 10675 morphological questions) [19].

We used WS353, SimLex-999, and UMNSRS for similarity calculations and Google Analogy for analogies, respectively.

### Graphical user interface

**Fig. 6:**
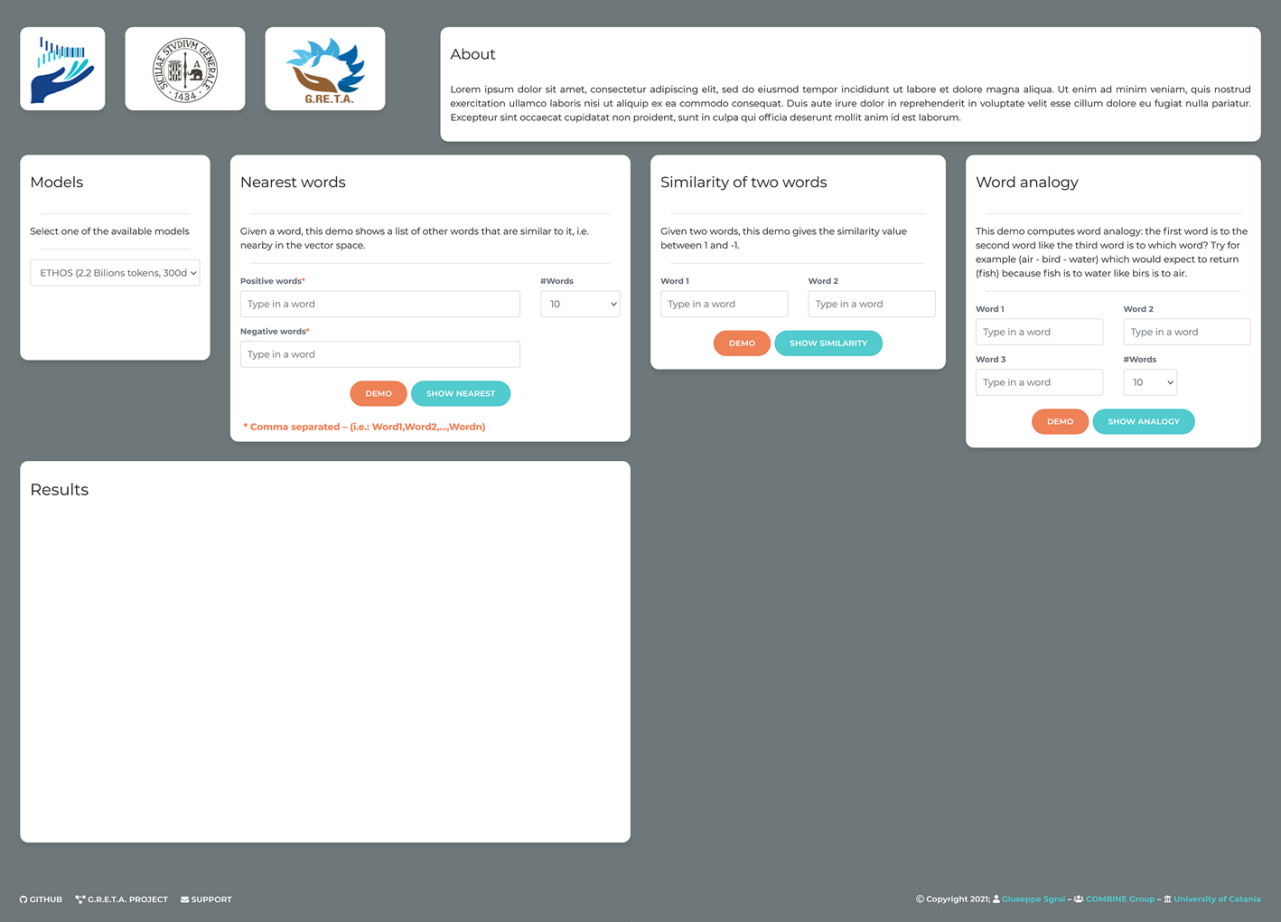
Web Graphic User Interface of ETHOS. This figure represents the GUI of ETHOS, which allows the execution of various analyses. In the center of the figure, the available analyses. On the left-hand side at the bottom of the figure is the table showing the analysis results

The Graphical User Interface (GUI) of ETHOS is fully developed using Python 3.9 programming language, the Django environment to create the web infrastructure (Fig. 6). It consists of five main boxes: i) Models, ii) Nearest Words, iii) Similarity of two words, iv) Word analogy, and v) Results.

Specifically:

- *Models* this box allows to load a model from those selected in memory. Among those available are: our model (ETHOS), PubMed [43], GloVe [44], and fastText [45]. In addition, other pre-trained models can be added manually.
- *Nearest words* given one or a set of words (positive and/or negative), separated by a comma, n-words are shown in such a way that they are similar to the input, i.e., near in vector space. In addition, it is possible to choose the number of words to be displayed (e.g., 5, 10, 25, 50, 100).
- *Similarity of two words* given two words as an input, the similarity score will be obtained. This value is between -1 and 1. If the score is equal to 1 the two words are identical (i.e., cancer-cancer = 1 or cancer-breast = 0.85).
- *Word analogy* this box allows to calculate the analogies between words (previously described). If the first word is to the second one as the third word is to which one? For example, if "Tumor" stands for "cancer" and "woman" stands for "breast", with 0.53 of similarity calculated with the cosine distance.
- *Results* the tabulated results obtained from the analyses are shown in this box.

## Results and discussion

In this section, we show the results obtained with the best model from the hyper-parameter step. More specifically, we evaluate the efficiency of the model in terms of standard metrics and the ability to obtain meaningful words according to the input used. First, we perform standard tests for similarity and analogy. As shown in Table 2, by performing the similarity tests we obtained a positive correlation with WS353 (Pearson: 0.34—Spearman: 0.37—OOV ratio: 51.71) and UMNSRS449 (Pearson: 0.62—Spearman: 0.69—OOV ratio: 91.31). While using the SimLex999 test (Pearson: 0.15—Spearman: 0.13—OOV ratio: 52.25) resulted in a very low correlation, therefore not significant. We can affirm that the model has succeeded in learning a clinical context than a generic one, just notice the high correlation obtained. Within the analogy test, we obtained a score of 0.23 using Google Analogy. More details can be found in the Additional file 1: supplementary data.

**Table 2:** Model performance

Metric	File	Method	Score
Similarity	WS353	Pearson correlation Spearman correlation OOV ratio	0.34 0.37 51.71
Similarity	UMNSRS449	Pearson correlation Spearman correlation OOV ratio	0.62 0.69 91.31
Similarity	SimLex999	Pearson correlation Spearman correlation OOV ratio	0.15 0.13 52.25
Analogy	Google Analogy	Accuracy	0.23

Metrics are listed, obtained using tests in the generic domain (WS353 and SimLex999) and clinical domain (UMNSRS449)

Out-of-vocabulary (OOV) is a metric usually expressed as a percentage, and represents the number of unknown terms that are not part of the normal lexicon found in a natural language processing environment [45]. When a word that’s not in the training set occurs in real data, this causes a problem. There are various techniques to avoid a zero-probability occurrence including smoothing and replacing the word a synonym.

Secondly, intrinsic tests were carried out on word embeddings across the n-grams created from the texts obtained from the survey. As shown in Table 3, the model extracts the context (words) owning high accuracy from the words that are nearest to the input. The tested n-grams consisted of unigrams, bigrams, trigrams and tetragrams. The scores awarded are contained in the csv files of the additional data.

**Table 3:** Nearest words results

Input	Nearest words
Age	Old, aged, woman, screen, screening
Comorbidities	Underweight, divorce, demographics, overweight, obese
Smoking	Diabetes, obesity, smoker, mellitus, hypertension
Diabetes	Mellitus, smoking, hypertension, obesity, smoker
Hypertension	Diabetes, mellitus, smoking, smoker, obesity
Alcohol	Consumption, obesity, drink, smoking, inactivity
Size	Tumor, small, large, location, diameter
Location	Quadrant, upper, size, medial, locate
Multicentricity	Multifocality, bilaterality, chinoy, vyas, mittra
Histology	Invasive, ductal, lobular, carcinoma, histological
Subtype	Luminal, intrinsic, basal, expression, molecular
Grade	Dcis, intermediate, high, histological, invasive
her2	Trastuzumab, triple, her2-, receptor, lapatinib
Stage	Early, patient, follow, breast, therapy
Multifocality	Multicentricity, multifocal, invasion, unifocal, lymphovascular
ki67	ki-67, her2, proliferation, labeling, expression
Palpable	Localization, lesion, wire, ultrasound, impalpable
Neoadjuvant	Chemotherapy, response, chemo-, therapy, adjuvant
Pain	Relief, symptom, neuropathic, persistent, severe
Breast, volume	Surgery, result, technique, patient, tissue
Patient, preference	Choice, option, undergo, need, surgery
Family, history	Genetic, hereditary, risk, mutation, brca1
Breast, density	Cancer, mammographic, woman, result, dense
Ovarian, cancer	Breast, woman, mutation, brca1, risk
Previous, radiotherapy	Radiation, postoperative, patient, result, surgery
Previous, scars	Scar, radial, histology, prior, type
Body, image	Sexuality, sexual, psychological, mass, attractiveness
Breast, ptosis	Result, surgery, ptotic, patient, technique
Breast, type	Cancer, patient, tumor, result, follow
Chest, wall	Thoracic, muscle, anterior, tissue, abdominal
Medical, history	Family, department, center, university, school
Skin, flaps	Flap, closure, nipple, reconstruction, lateral
Tissue, quality	Reconstruction, skin, life, technique, autologous
Health, insurance	Public, social, policy, state, healthcare
Locoregional, recurrence	Local, distant, survival, recur-, rence
Surgical, complications	Complication, procedure, surgery, technique, postoperative
Excision, rate	Local, margin, recurrence, follow, compare
Mastectomy, rate	Follow, patient, undergo, compare, year
Patients, satisfaction	Quality, life, outcome, psychological, psychosocial
Implant, loss	Reconstruction, expander, complication, extrusion, contracture
Local, recurrence	Distant, locoregional, survival, recur-, rence
Reconstruction, rate	Immediate, complication, mastectomy, follow, patient
Surgical, complication	Procedure, reconstruction, technique, surgery, immediate
Cosmetic, result	Outcome, surgery, good, follow, excellent
Overall, survival	Recurrence, difference, rate, disease, hazard
Previous, breast, surgery	Patient, result, follow, surgical, oncoplastic
Disease, free, survival	Overall, recurrence, distant, local, locoregional
Patient, reported, outcomes, measurements	Undergo, follow, rate, compare, report

For each word or pair of words in the n-grams (unigrams, bigrams, trigrams, and tetragrams), the five closest words obtained are listed

Finally, a set of word pairs was created to study their similarity. Table 4 shows all the pairs tested with our model.

**Table 4:** Similarity results

Word 1	Word 2	Score
Size	Volume	0.4693
Wish	Preference	0.3771
Desire	Preference	0.3064
Choice	Preference	0.5907
Profile	Profiling	0.2146
Treatment	Neoadjuvant	0.523
Primary	Neoadjuvant	0.547
Reconstructive	Reconstruction	0.572
Surgical	Surgery	0.6821
Radiation	Radiotherapy	0.7357
Result	Outcome	0.5993
Feeling	Outcome	0.1589

The similarity score was calculated for each pair of words

Experts have not received any feedback of the first round’s results yet. It is foreseen that they are going to receive a feedback before round 2, where consensus on the selected variables will be asked.

The model used for these tests is available in the Additional file 1: supplementary data on GitHub in Gensim and text format.

## Conclusions

The goal of NLP is to enable computers to communicate with humans in their own language, with the aim to make them capable of reading a text, listening to a voice, interpreting it, measuring sentiment (through 'sentiment analysis') and determining what content is the most meaningful. Specifically, these algorithms were created to analyze the grammar and identify the rules of natural language. The ambiguity and peculiar characteristics of the NLP technique make this process articulated and complex. This study allows experts to reconstruct a context from a text and generate new hypotheses or "variables" that could be studied later. Therefore, natural language processing also allows automatic and efficient management of document classification through the extraction of the information contained in the documents.

The following goals are to extend the training dataset with new texts (papers and books) to improve the final output, use new neural network models like Doc2Vec, add representations through Principal Component Analysis (PCA), and extend the GUI with new features. Finally, the ability to download results from the GUI in one of the standard formats (e.g., CSV or JSON) will be added.

### Availability and requirements

- Project name: ETHOS-Word-Embeddings.
- Project home page: https://github.com/Pex2892/ETHOS-Word-Embeddings
- Operating system(s): Platform independent.
- Programming language: Python 3.
- Other requirements: none.
- Any restrictions to use by non-academics: not applicable.

## Supplementary Information

**Additional file 1.** Results (word pairs) of hyper-parameters combinations.

## Data Availability

The web-interface is fully described in the paper and is available at: https://combine.dmi.unict.it/ETHOS-word-embeddings/.

## Abbreviations

PWC: PricewaterhouseCoopers
BCS: Breast cancer surgery
RCTs: Randomized Controlled Trials
HER: Health electronic record
NLP: Natural language processing
ML: Machine learning
AI: Artificial Intelligence
GPT-3: Generative pre-trained transformer 3
G.RE.T.A.: Group for Reconstructive and Therapeutic Advancements
ETHOS: FEatures TecHniques Outcomes Survey
DC: Data cleaning
POS: Part-of-speech
W2V: Word2Vec
CBOW: Continuous bag of words
SG: Skip-gram
HS: Hierarchical softmax
WS353: WordSim-353
RW: Rare word
GUI: Graphical user interface
OOV: Out-of-vocabulary
PCA: Principal component analysis

## Footnotes

1 https://projectredcap.org
