# Supplementary material for: Evaluation of word embedding models to extract and predict surgical data in breast cancer
Source: BMC Bioinformatics. 2022 Nov 16;22(Suppl 14):631. doi: 10.1186/s12859-022-05038-6 (PMC9667561; doi:10.1186/s12859-022-05038-6)
Supplement: Supplementary file 1 — Additional file 1. Results (word pairs) of hyper-parameters combinations. [file 12859_2022_5038_MOESM1_ESM.zip › New folder/Supplementary.docx]

**Titolo**

Autori

- The "ETHOS_model_metrics.tsv" (tab-separated values) file contains the performance obtained by testing the model.
- The "ETHOS_vectors.txt" file is the model in text format.
- The "ETHOS.model" file is the model in gensim format.
- The "evaluators" folder contains the evaluators used to test the model:
  - WS353.tsv (similarity)
  - SimLex999.tsv (similarity)
  - UMNSRS449.tsv (similarity)
  - questions-words.tsv (analogy)
- The excel file "Hyper-parameters_model.xlsx" contains all models tested during the Hyper-parameters phase. The best one obtained is called Test16 which coincides with the file "ETHOS.model".
- The "input" folder contains the n-grams and similarity files respectively.
- The "output" folder contains the files obtained from the analyses for n-grams (nearest words) and similarity (word pairs) respectively.
